# Empirical mode decomposition with missing values

**DOI:** 10.1186/s40064-016-3692-1

**Published:** 2016-11-25

**Authors:** Donghoh Kim, Hee-Seok Oh

**Affiliations:** 1 Department of Applied Mathematics, Sejong University, Seoul, 05006 Korea; 2 Department of Statistics, Seoul National University, Seoul, 08826 Korea

**Keywords:** Empirical mode decomposition, Imputation, Missing, Multiscale method, Self-consistency

## Abstract

This paper considers an improvement of empirical mode decomposition (EMD) in the presence of missing data. EMD has been widely used to decompose nonlinear and nonstationary signals into some components according to intrinsic frequency called intrinsic mode functions. However, the conventional EMD may not be efficient when missing values are present. This paper proposes a modified EMD procedure based on a novel combination of empirical mode decomposition and self-consistency concept. The self-consistency provides an effective imputation method of missing data, and hence, the proposed EMD procedure produces stable decomposition results. Simulation studies and the image analysis demonstrate that the proposed method produces substantially effective results.

## Background

Multiscale methods for decomposing a signal into several significant modes with simple forms have been widely studied for last two decades. Since the decomposition procedure reduces the complexity of a signal and at the same time, enhances interpretability of its components, the information embedded in a signal can be easily recovered.

Spectral analysis (Priestley 1981) and wavelet analysis (Mallat 2009; Daubechies 1992; Vidakovic 1999) are popular multiscale methods for signal decomposition. Huang et al. (1998) proposed a data-adaptive procedure called empirical mode decomposition (EMD), and Daubechies et al. (2011) proposed an alternative method of EMD, termed synchrosqueezed wavelet transforms, which are based on reassignment methods of wavelet coefficients. These multiscale decomposition methods implicitly assume that a signal is observed at equally spaced time points. Since the local behavior of a signal can evolve over time, by utilizing the local information observed at equally spaced time points, it is useful for identifying the amount of variation at different scale and time location and for extracting each superimposed component. However, for many signals, missing values occur quite common. In practice, large amount of missing values may occur at random, for example, for intermittent wireless signal caused by malfunction of network device for one-dimensional signal and partial fingerprint due to incomplete touch in digital scanner for two-dimensional image. The problem we concern in this paper is that when some observations are missing, most multiscale decomposition methods produce ineffective outcome. Especially, since EMD depends on the behavior of local extrema, missing of local extrema causes severe distorted results. The brief review of EMD procedure will reveal this aspect more clearly.

 Huang et al. (1998) proposed a data-driven multiscale procedure for sequentially separating each superimposed component from a given signal *s*(*t*) as follows. First, identify local extrema and construct two functions called upper and lower envelopes by interpolating local maxima and local minima, respectively. Second, take a mean of upper and lower envelopes, which produces a signal with a lower frequency than that of the original signal *s*(*t*). Third, subtract the mean envelope from the signal *s*(*t*) and obtain a highly oscillatory wave *h*(*t*). Such an oscillatory wave *h*(*t*) is defined as an intrinsic mode function (IMF) if it satisfies two conditions: (1) the number of extrema and the number of zero-crossings of *h*(*t*) is equal or differ by one and (2) the local average of *h*(*t*) is zero. If the wave *h*(*t*) does not satisfy the above conditions, then the same procedure is repeated for *h*(*t*) until the conditions are satisfied. This iterative process is called sifting. Through sifting the original signal *s*(*t*) is decomposed into the highest frequency ${{\mathrm{imf}}}_1(t)$ and a residual signal $r_1(t) = s(t) - {{\mathrm{imf}}}_1(t)$. As the sifting is applied to the residue, the signal is sequentially decomposed into several signals having different frequencies from the highest-frequency component ${\mathrm{imf}}_1(t)$ to the lowest-frequency component ${\mathrm{imf}}_n(t)$ and a residual signal *r*(*t*). After decomposition, we finally have *n* IMFs and a residual signal

$$\begin{aligned} s(t) = \sum _{i=1}^{n} {\mathrm{imf}}_i(t) + r(t). \end{aligned}$$

In the literature, there have been many studies to enhance the performance of the conventional EMD. Boudraa and Cexus (2007) separated the high-frequency components using a filtering method. Wu and Huang (2009) developed the ensemble EMD (EEMD) by averaging the simulated signals. The variants of EEMD have been proposed by several authors. The complementary ensemble EMD (CEEMD) (Yeh et al. 2010) was introduced by adding pairs of positive and negative noises into a signal and applying EEMD. Torres et al. (2011) proposed the complete ensemble EMD with adaptive noise (CEEMDAN). EEMD is applied to each stage of decomposition by adding a noise to a signal and a residue after each IMF extraction. The improved complete ensemble EMD (ICEEMD) (Colominas et al. 2014) controlled noise level between the added noise and a residue for CEEMDAN process. Xu et al. (2009) proposed a hybrid extrema estimation algorithm based on Fourier interpolation to decompose signals with lower sampling rate. Diop et al. (2010) suggested a PDE-based approach to compute envelopes. Barnhart et al. (2011, 2012) provided a methodology for discontinuous data by applying EMD on each individual continuous data segment, and by adapting mirroring approach for the discontinuous data gaps. Kim et al. (2012a) introduced the statistical EMD adapting smoothing of local extrema instead of interpolation. Komaty et al. (2014) suggested a signal-filtering approach based on a combination of EMD and a similarity measure for noise removal. Park et al. (2015) applied a quantile smoothing method to a signal itself instead of interpolating local extrema of a signal for sifting. The extension of EMD to two-dimensional image has been developed by several authors. Damerval et al. (2005) employed moving window and Nunes et al. (2005) used morphological operation for two-dimensional extrema detection. Bhuiyan et al. (2008) proposed order-statistics filter method for envelope estimation of two-dimensional image. Kim et al. (2012b) proposed a two-dimensional EMD through the smoothing sifting of two-dimensional local extrema.

As observed in the aforementioned EMD procedure, when some missing values are present, EMD produces distorted decomposition results due to two reasons: (1) when the observations are not equally spaced, it is difficult that the local behavior of a signal can be captured in a balanced way and (2) especially if missing occurs in local extrema, the sifting fails to capture upper and lower envelopes properly.

**Fig. 1:**
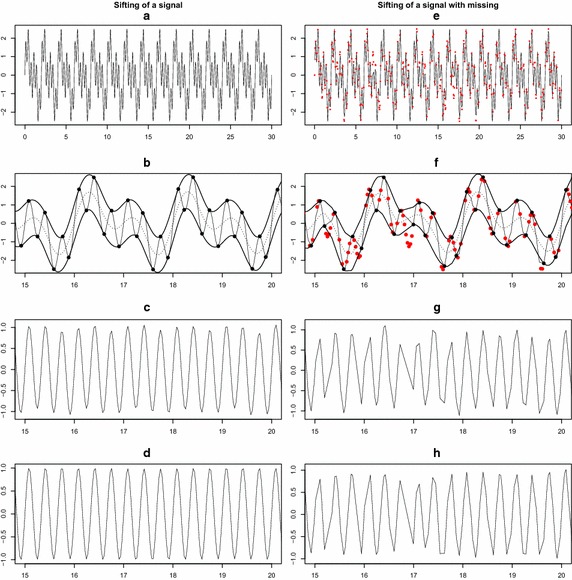
Sifting process for a signal with 40% missing values. **a** Signal, **b** extrema and envelopes, **c** candidate h, **d** $\mathrm{imf}_1$, **e** signal with missing, **f** extrema and envelopes, **g** candidate h, **h** $\mathrm{imf}_1$

We consider a simulated example that clarifies the above-mentioned problem of the conventional EMD and provides a motivation of the proposed method. The left panel of Fig. 1 illustrates a signal of three components $\sin (\pi t)+ \sin (2 \pi t) + \sin (6 \pi t)(t \in [0, 30])$ and sifting process. Figure 1b shows upper and lower envelopes constructed by interpolating the local maxima and minima (black points), respectively, and its mean envelope denoted by dotted line on $t \in [15, 20]$. By subtracting the mean envelope from the original signal, a candidate IMF *h* is obtained as in Fig. 1c. The sifting process continues until the first IMF $imf_1$ in Fig. 1d is extracted. On the other hand, the right panel of Fig. 1 shows the sifting process for a signal with 40% missing values denoted by red points. By observing the location of local extrema and missing values in Fig. 1e, we notice that the overall pattern of the original signal cannot be captured between two envelopes. Especially, on the area around $t=17$ in Fig. 1f, the lower envelope is distorted due to the missing values. This phenomena eventually produces improper candidate IMF *h* and the first IMF $imf_1$ as displayed in Fig. 1g, h. Thus, the conventional EMD does not work properly to decompose the three component signal with missing values, and hence, fails to extract the sinusoid component $\sin (6 \pi t)$ effectively.

To improve EMD algorithm in the presence of missing values, we propose a new method by adapting the concept of self-consistency that recursively imputes missing values and decomposes the imputed signal efficiently under EMD framework. For practical implementation, we provide a modified EMD algorithm which consists of two alternating steps, imputation and decomposition. In addition, we discuss some remarks of the algorithm such as the fitting method, the selection of smoothing parameter in the fitting method, the choice of initial values, and so on. Furthermore, we extend the proposed method to a two-dimensional signal with missing values, so that this extension provides a meaningful influence on image decomposition.

The rest of the paper is organized as follows. In “Methods” section, we briefly review the self-consistency principle, and propose a new method for signal decomposition in presence of missing values with a practical algorithm. To evaluate empirical performance of the proposed method, simulation studies for one-dimensional signal are conducted in “Numerical study” section, and a real data example is presented in this section. Furthermore, in “Extension to image” section, the extension to two-dimensional signals is discussed. Lastly, conclusions are addressed in “Conclusions” section.

## Methods

### Review: self-consistency

Tarpey and Flury (1996) introduced the self-consistency as a fundamental concept in statistics, which is inspired by Hastie and Stuetzle (1989) for developing principal curves.

#### **Definition 1**

(Tarpey and Flury 1996) A random signal *f* is *self-consistent* for *g* if $E(f|g)=g$ almost surely.

As pointed out in Tarpey and Flury (1996), there is a close connection between the concept of self-consistency and Expectation-Maximization (EM) algorithm of Dempster et al. (1977). The EM algorithm generates a sequence of self-consistent random variables and the maximum likelihood estimator satisfies the self-consistency condition. Further, Lee and Meng (2007) considered a self-consistent regression estimator with incomplete data. They proposed an estimate ${\hat{f}}_{obs}$ of *f* given observed data ${\varvec{X}}_{obs}$ that is the solution of the following self-consistent equation

1 $$\begin{aligned} E({\hat{f}}_{com}|{\varvec{X}}_{obs},f={\hat{f}}_{obs})={\hat{f}}_{obs}, \end{aligned}$$

where ${\hat{f}}_{com}$ denotes an estimate of *f* based on the imaginary complete data ${\varvec{X}}_{com}=({\varvec{X}}_{obs},{\varvec{X}}_{mis})$ and ${\varvec{X}}_{mis}$ is missing data. Since Eq. (1) does not depend on the method for estimation, it can be applicable for EMD approach with missing data.

### Proposed algorithm

Suppose that a signal *s*(*t*) is observed at equally spaced time points, but there are some missing values at a set of time points $t_m$. Let $t_c=(t_o,t_m)$ be the set of time points in the complete data $s(t_c)$, where $t_o$ denotes the set of time points for the observed signal. Thus, it follows that $s(t_c)=(s(t_o),s(t_m))$. Suppose for the moment that, given $s(t_c)$, we have a decomposition by EMD as $s(t_c)= \sum\nolimits_{i=1}^{n} {\mathrm{imf}}_{i}(t_c) + r(t_c)$. In practice, the values $s(t_m)$ are not available, and hence, given imputed values ${\hat{s}}(t_m)$, we consider an estimated decomposition as

2 $$\begin{aligned} {\hat{s}}(t_c)= (s(t_o),{\hat{s}}(t_m))=\sum _{i=1}^{n} {{\widehat{{\mathrm{imf}}}}}_{i}(t_c) + {\hat{r}}(t_c). \end{aligned}$$

Thus, it is required to have a method to fit the data at the observed locations and predict ${\hat{s}}(t_m)$ at the missing locations, which are used for imputed values.

For this purpose, we consider the self-consistent Eq. (1) under this framework as

3 $$\begin{aligned} E({\hat{f}}_{com}|s(t_o),{\hat{f}})={\hat{f}}. \end{aligned}$$

Suppose that ${\hat{f}}(\cdot )$ satisfies the above equation. Then the imputed values can be obtained by ${\hat{s}}(t_m):={\hat{f}}(t_m)$, and hence, we finally obtain the decomposition in (2). In fact, the rationale to employ Eq. (3) for our missing problem can be explained as follows. Let ${\hat{f}}_{com}$ be the estimator based on the complete data and ${\hat{f}}$ be the estimator based on the observed data. For any ${\hat{f}}$ and a set of missing time points $t_m$, it follows that, under the assumption that the missing pattern is random,

$$\begin{aligned} \Vert s(t_m)-{\hat{f}}(t_m)\Vert ^2 &= \Vert s(t_m)-{\hat{f}}_{com}(t_m)+{\hat{f}}_{com}(t_m)-{\hat{f}}(t_m)\Vert ^2 \\ &= \Vert s(t_m)-{\hat{f}}_{com}(t_m)\Vert ^2+\Vert {\hat{f}}_{com}(t_m)-{\hat{f}}(t_m)\Vert ^2\\&= \Vert s(t_m)-{\hat{f}}_{com}(t_m)\Vert ^2+\Vert {\hat{f}}_{com}(t_m)-E[{\hat{f}}_{com}(t_m)|s(t_o),{\hat{f}}(t_m)]\Vert ^2\\&\quad +\,\Vert E[{\hat{f}}_{com}(t_m)|s(t_o),{\hat{f}}(t_m)]-{\hat{f}}(t_m)\Vert ^2. \end{aligned}$$

Hence, the minimization of $\Vert s(t_m)-{\hat{f}}(t_m)\Vert ^2$ over ${\hat{f}}$ at the set $t_m$ is equivalent to minimization of $\Vert E[{\hat{f}}_{com}(t_m)|s(t_o),{\hat{f}}(t_m)]-{\hat{f}}(t_m)\Vert ^2$, which is obtained by the solution of (3).

Here we propose a simple and fast algorithm to implement (2) and (3) as follows. Given initial values ${\hat{s}}^{(0)}(t_m)$,

1. Iterate, until convergence, the following alternating steps for $\ell =1,2,\ldots ,$
  1.1. Imputation Step: Fit ${\hat{f}}^{(\ell -1)}(t_o)$ at the observed locations $t_o$, impute by prediction ${\hat{s}}^{(\ell )}(t_m):={\hat{f}}^{(\ell -1)}(t_m)$ at the missing locations $t_m$ and construct a complete data ${\hat{s}}^{(\ell )}(t_c):=(s(t_o),{\hat{s}}^{(\ell )}(t_m))$.
  1.2. Decomposition Step: Apply EMD procedure to ${\hat{s}}^{(\ell )}(t_c)$ and obtain
    $$\begin{aligned} {\hat{s}}^{(\ell )}(t_c)=\sum _{i=1}^{n} {\widehat{{\mathrm{imf}}}}_{i}^{(\ell )}(t_c) + {\hat{r}}^{(\ell )}(t_c). \end{aligned}$$
  1.3. The iteration stops if $\frac{||{\hat{s}}^{(\ell + 1)}-{\hat{s}}^{(\ell )}||}{||{\hat{s}}^{(\ell )}||} \le \delta$ for some tolerance level $\delta > 0$.
2. Take the converged IMFs as the final IMFs.

We have some remarks regarding the aforementioned algorithm:

- For the fitting method, we consider various nonparametric function estimation methods such as smoothing splines, kernel smoothing, and the local polynomial regression. The asymptotic results of the equivalent kernel described in Silverman (1984) support the fact that both a spline-type estimator and kernel smoother including local polynomial regression estimator can be written
  $$\begin{aligned} {\hat{f}}(t)=\frac{1}{n}\sum _{i=1}^nw(t,t_i)s(t_i), \end{aligned}$$
  where $w(t,t_i)$ denote weights at time point $t_i$. In this study, we employ the smoothing splines with a smoothing parameter chosen by generalized cross-validation.
- In this study, we use the local mean values as ${\hat{s}}^{(0)}(t_m)$ for the choice of initial values.
- Note that the imputation step does not depend on the dimension of *s*. Thus it can be easily extended to two-dimensional image. The only modification required is to replace the 1-dimensional smoothing method in the imputation step by a 2-dimensional method such as thin-plate smoothing splines.

## Numerical study

### Simulation study

Here we discuss results from a simulation study that was designed to assess the practical performance of the proposed method. In this simulation study, we compare the proposed method with two other methods:

1. EMD.obs: the conventional EMD algorithm with observed data,
2. EMD.self: the proposed EMD algorithm described in “Proposed algorithm” section, and
3. EMD.com: the conventional EMD algorithm with imaginary complete data.

**Fig. 2:**
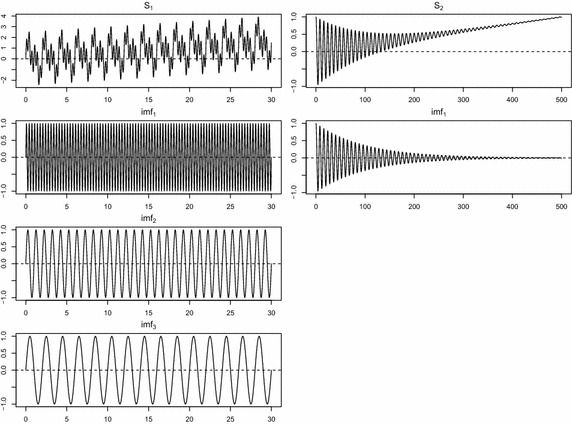
Test functions

Note that EMD.com is used as a benchmark for comparison. We consider two test functions, (1) a composite sine function of the form $S_1(t)= \sin (\pi t)+ \sin (2 \pi t) + \sin (6 \pi t) + 0.05t(t \in [0, 30])$, and (2) a chirp signal of the form $S_2(t) = \exp (-0.01t) \cos (\pi t/4)+0.002t(t \in [0, 500])$. Figure 2 displays two test functions and their components.

To evaluate how the proposed algorithm performs according to missing data percentage, we consider five different missing percentages: 10, 20, 30, 40 and 50%. In addition, we consider two cases of missing pattern: (a) The first one is missing at random where missing locations were randomly selected from inside 90% of the time domain and missing values do not exist near boundaries. (b) The second one is missing at random where missing locations were randomly selected over the entire domain including boundaries.

For each test function, missing percentage, and missing pattern, 100 datasets with sample size 2000 are generated. For each generated dataset, three methods above were applied to decompose the test function. To evaluate the decomposition results, we consider mean squared error (MSE) as a discrepancy measure

$$\begin{aligned} \text{ MSE }(g(t_o),{\hat{g}}(t_o))=\frac{1}{{\#} \{t_o\}}\sum _{t_i \in t_o} \{g(t_i)-{\hat{g}}(t_i)\}^2, \end{aligned}$$

where *g* and ${\hat{g}}$ denote the true component and the corresponding extracted IMF.

**Fig. 3:**
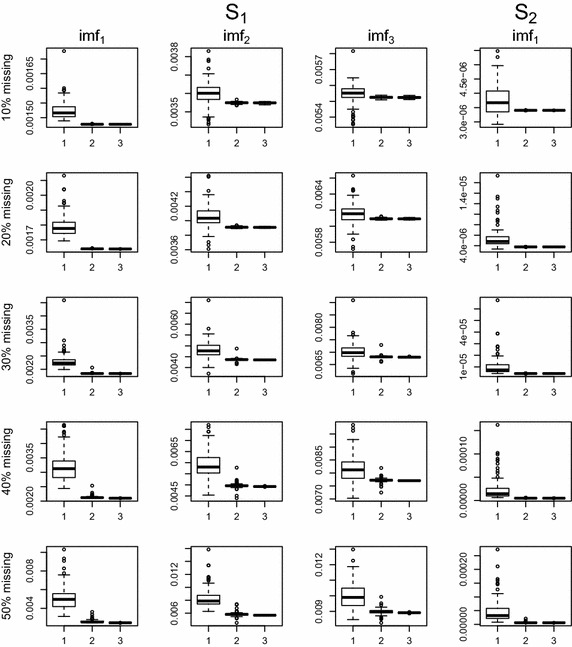
Boxplots of MSE values according to missing percentages, 10, 20, 30, 40 and 50% when missing does not exists near the boundary: *1* EMD.obs, *2* EMD.self, *3* EMD.com

**Fig. 4:**
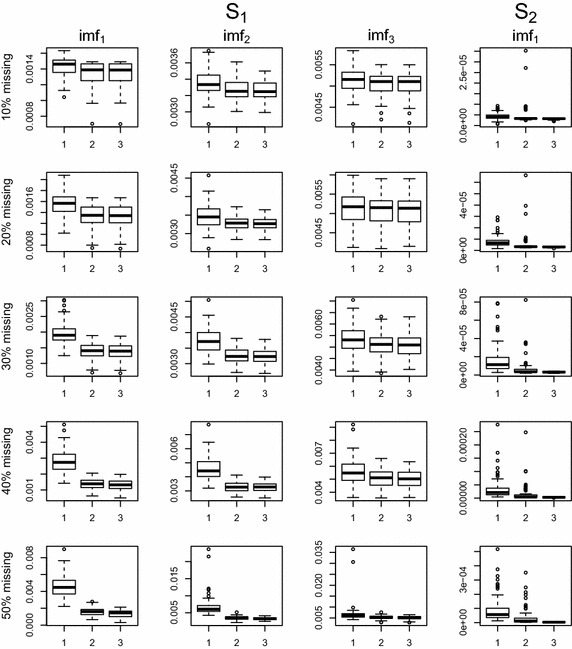
Boxplots of MSE values according to missing percentages, 10, 20, 30, 40 and 50% when missing are spread over whole domain: *1* EMD.obs, *2* EMD.self, *3* EMD.com

Figures 3 and 4 show box plots of MSE values for two missing patterns. The proposed EMD.self is comparable to EMD.com when the missing data percentage is up to 50%. Even when missing exists near the boundary, EMD.self works well due to effective imputation by the proposed algorithm.

**Fig. 5:**
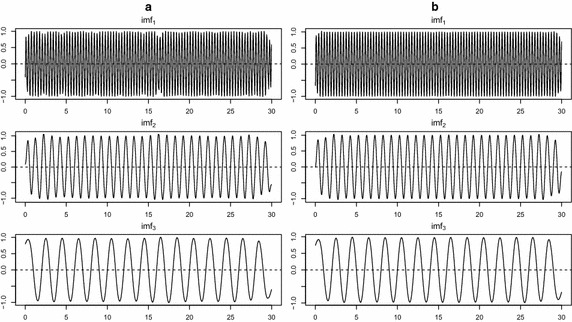
Comparison between decomposition results of noise-free signal $S_1$ by EMD.obs and EMD.self when missing percentage is 40%. **a** Decomposition by EMD.obs, **b** decomposition by EMD.self

**Fig. 6:**
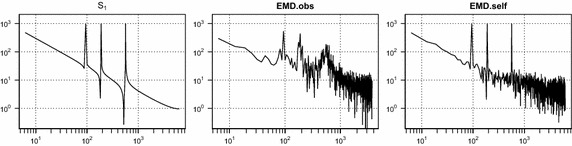
Periodogram of signal $S_1$, reconstruction by EMD.obs and reconstruction by EMD.self when missing percentage is 40%

**Fig. 7:**
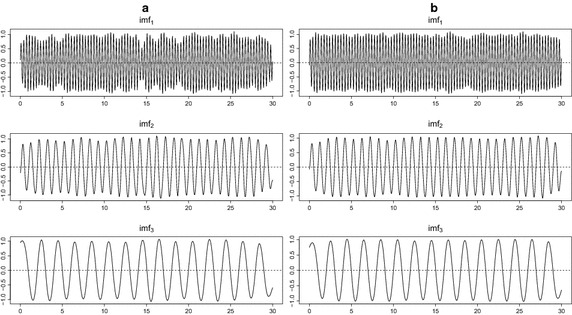
Comparison between decomposition results of noisy signal $S_1$ by EMD.obs and EMD.self when missing percentage is 40%. **a** Decomposition by EMD.obs, **b** decomposition by EMD.self

To access the property of the extracted IMFs, we compare the decomposition results for test signal $S_1$ with 40% missing values. Figure 5 illustrates that the decomposition result of the panel (b) by the proposed EMD.self produces more accurate IMFs than the decomposition result of the panel (a) by EMD.obs. Figure 6 shows that the periodogram of signal $S_1$, reconstruction by EMD.obs and reconstruction by EMD.self. The periodogram by EMD.self effectively detects three main frequencies of signal $S_1$, while it is difficult to identify three main frequencies from the periodogram by EMD.obs. The proposed method can be applied to a noisy signal. From Fig. 7 of noisy signal $S_1$ with the signal-to-noise ratio (SNR) seven, the proposed EMD.self produces more accurate IMFs than EMD.obs does. Here, SNR is defined as $||S_1||/ \sigma$ where $\sigma$ is the standard deviation of noise. Table 1 lists MSE values for noise-free signal $S_1$ in Fig. 5 and noisy signal $S_1$ in Fig. 7 by EMD.obs and EMD.self. As one can see, MSEs of extracted IMFs by the proposed EMD.self are smaller than MSEs by EMD.obs, for both noise-free and noisy signal.

**Table 1:** MSE values for noise-free signal $S_1$ in Fig. 5 and noisy signal $S_1$ in Fig. 7 by EMD.obs and EMD.self when missing percentage is 40%

	$imf_1$		$imf_2$		$imf_3$	
	EMD.obs	EMD.self	EMD.obs	EMD.self	EMD.obs	EMD.self
Noise-free $S_1$	0.0030	0.0010	0.0035	0.0026	0.0045	0.0040
Noisy $S_1$	0.0134	0.0061	0.0141	0.0071	0.0095	0.0052

**Fig. 8:**
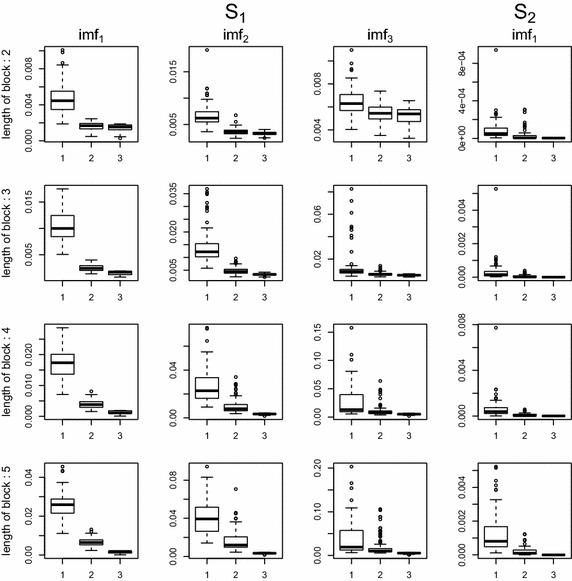
Boxplots of MSE values according to the length of block, 2, 3, 4 and 5 when missing percentage is 30%: *1* EMD.obs, *2* EMD.self, *3* EMD.com

Furthermore, it is common that the missing locations occur in consecutive time points, for example, due to the malfunction of sensor to measure a signal. As for the third missing pattern, we consider the missing occurs in consecutive time points to form a block of missing values. Box plots of MSEs according to different length of block are given in Fig. 8 when missing percentage is 30%. From Fig. 8, the proposed method is comparable to EMD.com when the length of block is up to four.

### Real data example

**Fig. 9:**
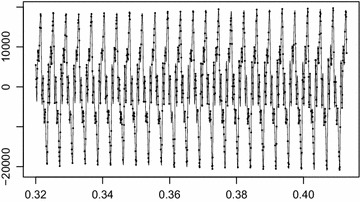
Clarinet sound with 40% missing values denoted by black points

**Fig. 10:**
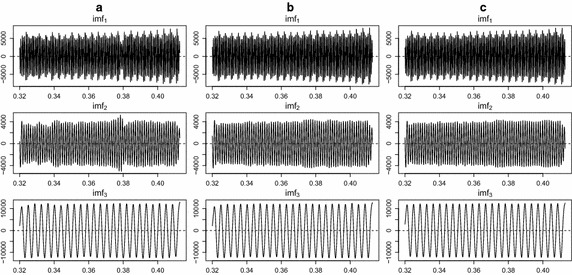
Decomposition of clarinet sound. **a** Decomposition of observed signal, **b** decomposition by the modified EMD, **c** decomposition of complete signal

The signal in Fig. 9 shows clarinet sound playing the note Coctave0. The data are available in the website http://wiki.laptop.org/go/Sound_samples. We extracted 2048 samples between 0.32 and 0.41 s with 22,050 Hertz for the analysis. For comparison, we intensionally make 40% missing values from clarinet signal denoted by black points. Then we perform the aforementioned three methods for decomposition. As shown from Fig. 10, the conventional EMD does not work properly to decompose a signal under the presence of missing values especially around 0.38 s, while the proposed method produces almost the same decomposition result to the decomposition result of the complete signal.

## Extension to image

Bidimensional EMD for two-dimensional signals such as images has been proposed by some studies (Damerval et al. 2005; Nunes et al. 2005; Bhuiyan et al. 2008; Kim et al. 2012b). To construct the upper and lower envelopes of two-dimensional signals, interpolation is done with the scattered sparse extrema. Therefore, missing aggravates insufficient sampling rate, and causes more obstacle to estimate candidate IMFs. Two-dimensional extension is straightforward by recursively imputing the two-dimensional missing values through thin-plate spline and decomposing imputed image by an existing two-dimensional EMD procedure.

Now we consider a test image *f* as, for $x, y \in [0,1]$,

$$\begin{aligned} f(x, y) = 2xy + \sin (15\pi x)\sin (15\pi y) + \sin (7\pi x)\sin (7\pi y). \end{aligned}$$

**Fig. 11:**
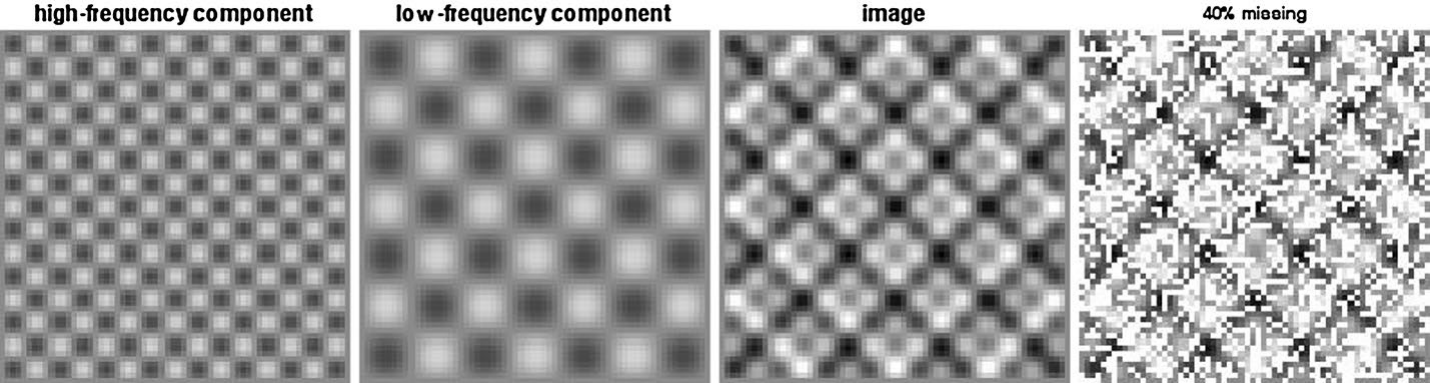
The test image

This image consists of two sinusoidal types of components and a trend component. Figure 11 shows test image *f*, which consists of the high-frequency component $\sin (15\pi x)\sin (15\pi y)$ and the low-frequency component $\sin (7\pi x)\sin (7\pi y)$. The right-most panel of Fig. 11 illustrates the image with 40% missing values. The white pixels represent the locations of the missing observations.

**Fig. 12:**
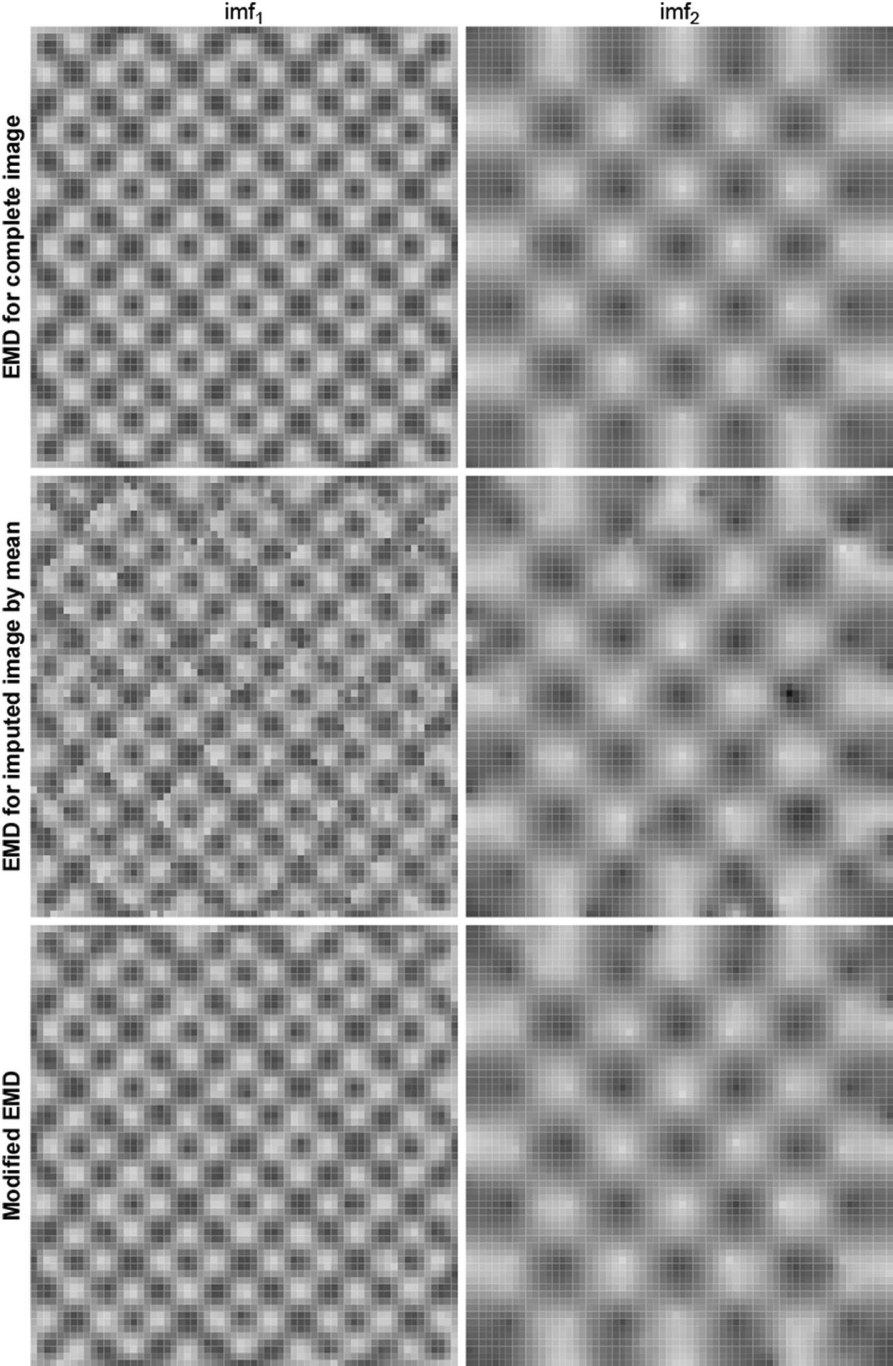
Decomposition results for the test image

To evaluate the proposed method, we compare the decomposition results for the complete image and imputed image with local mean near missing. The first and second rows of Fig. 12 represent two components as the decomposition results for the complete image and imputed image with local mean near missing. As shown, the proposed method extracts the proper IMFs, and decomposes the high-frequency and low-frequency components effectively as the decomposition for the complete image does. However the decomposition for the imputed image by mean does not extract the first IMF properly, which affects the subsequent decomposition results. There are lots of spots in the first IMF due to the improper imputation.

## Conclusions

In this paper, we have proposed a modified empirical mode decomposition to deal with missing data problems. The proposed method is based on imputation using the self-consistency principle. We have presented an effective algorithm for implementation of the proposed method. The empirical performance of the proposed method has been evaluated throughout various numerical experiments including both one and two-dimensional settings. Results from these experiments illustrate the proposed method possesses good empirical properties.
